# Supplementary material for: Enhancement of in vivo targeting properties of ErbB2 aptamer by chemical modification
Source: PLoS One. 2023 Sep 20;18(9):e0291624. doi: 10.1371/journal.pone.0291624 (PMC10511116; doi:10.1371/journal.pone.0291624)

Figure 2A Original image

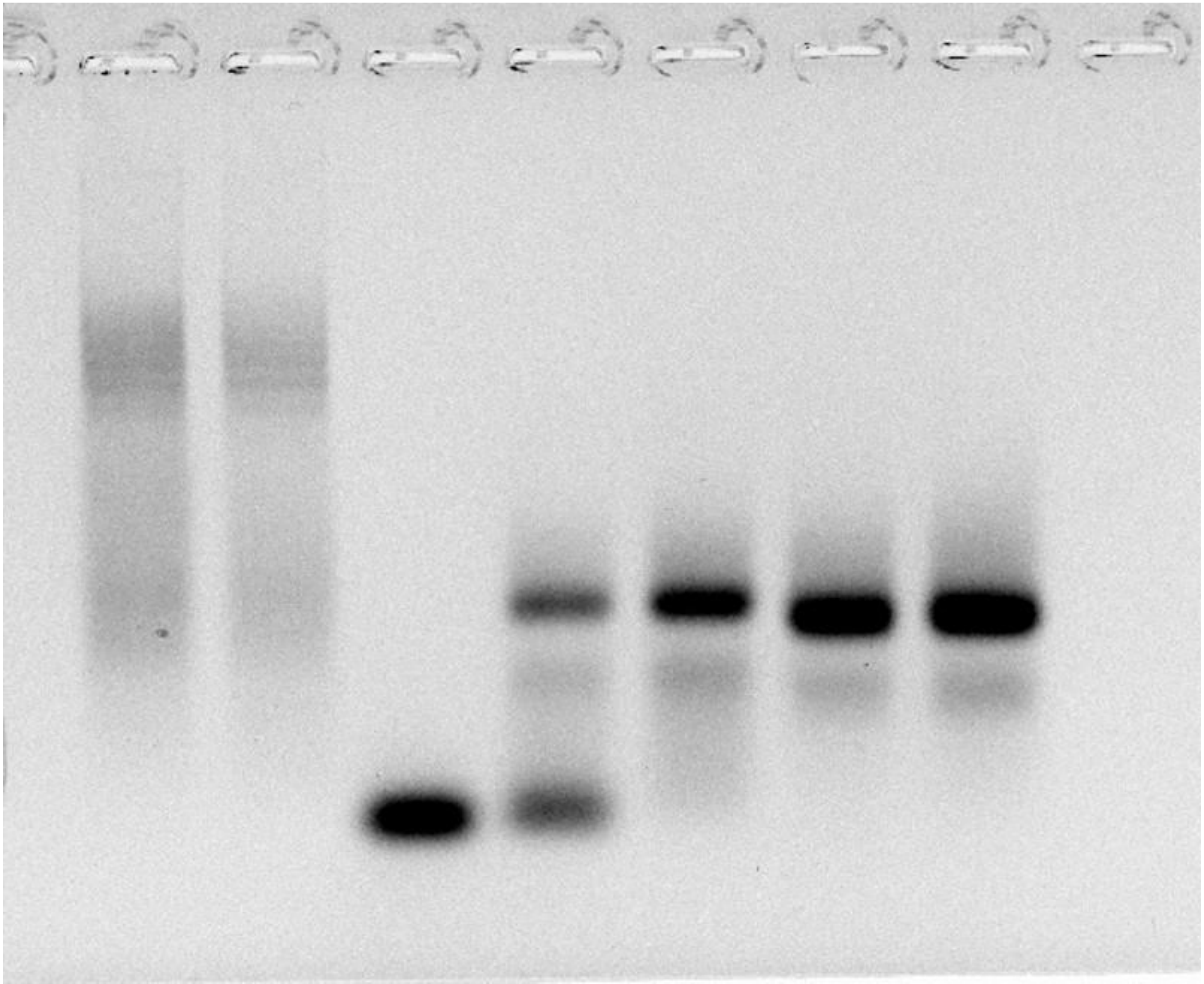

Figure 2A Annotated image

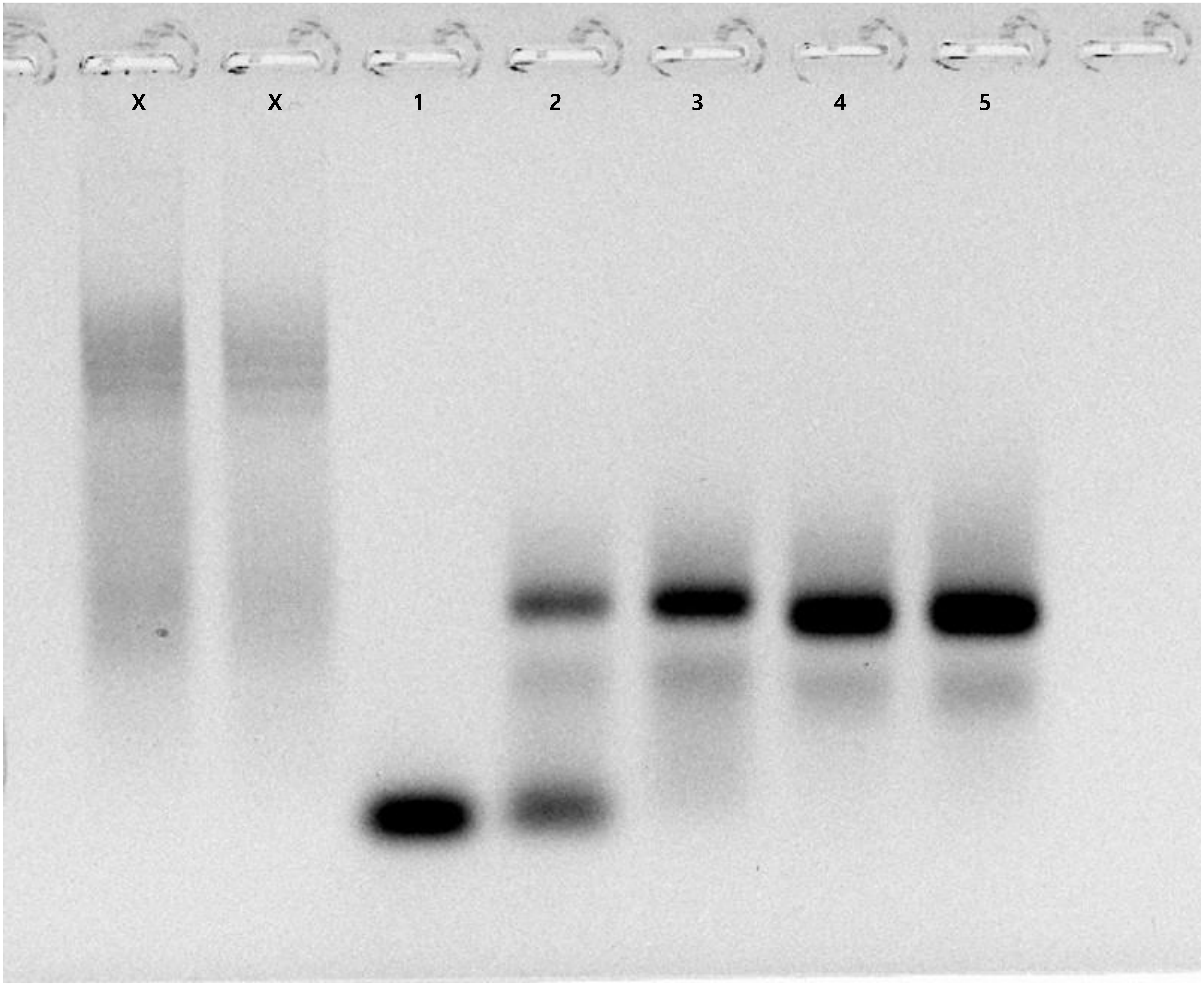

Figure 2B Original image of ErbB2

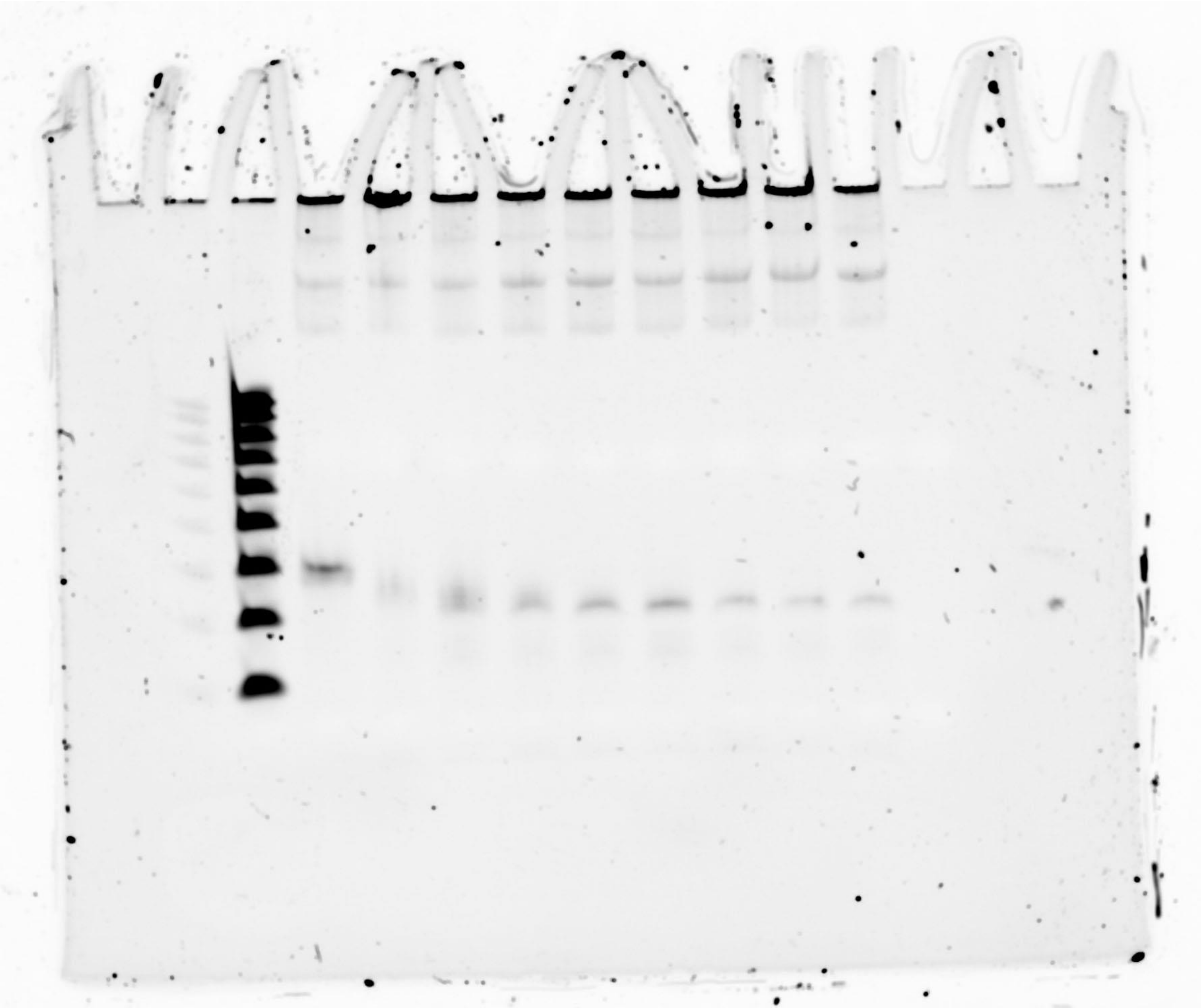

Figure 2B Annotated image of ErbB2

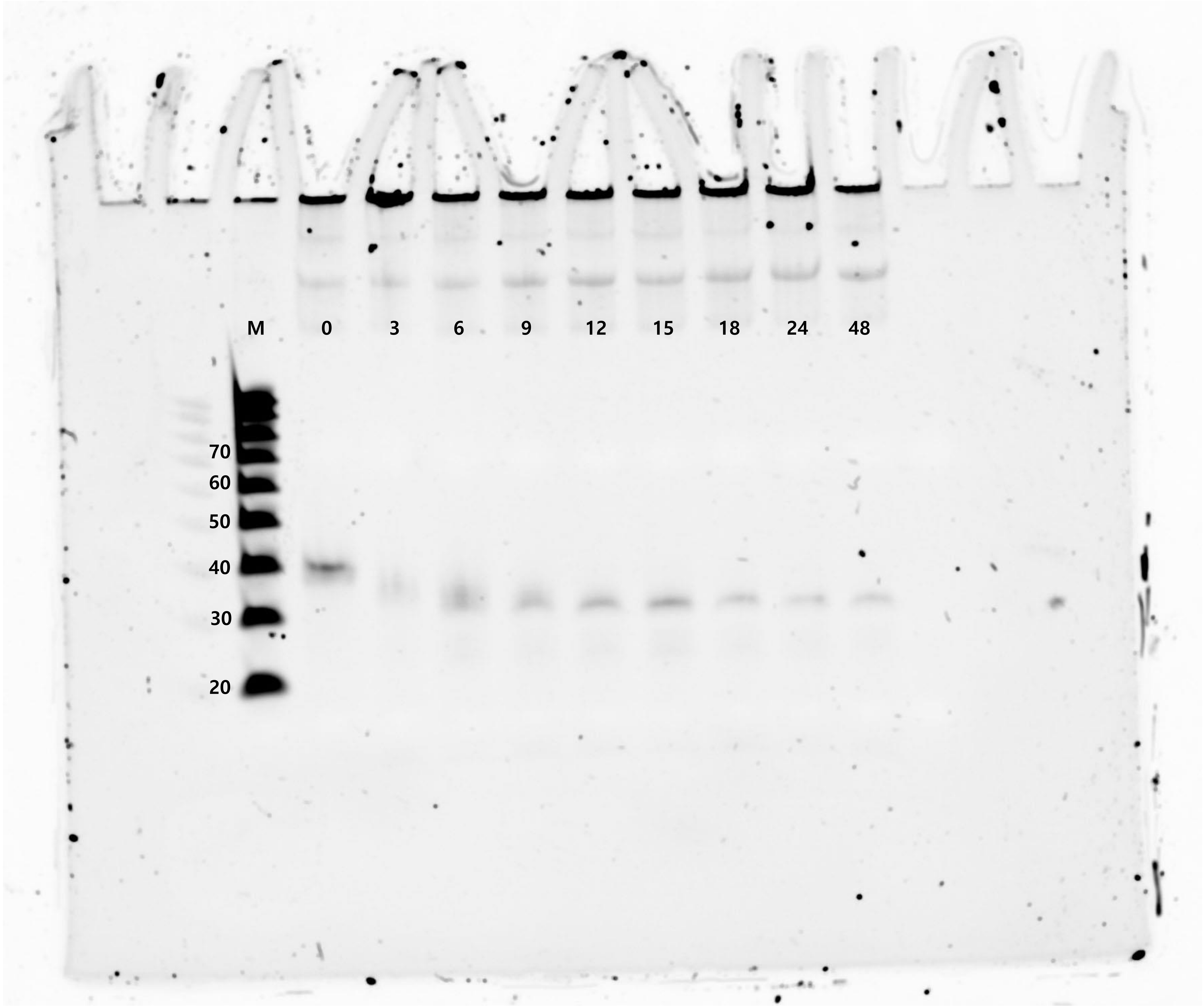

Figure 2B Original image of RC-ErbB2-idT

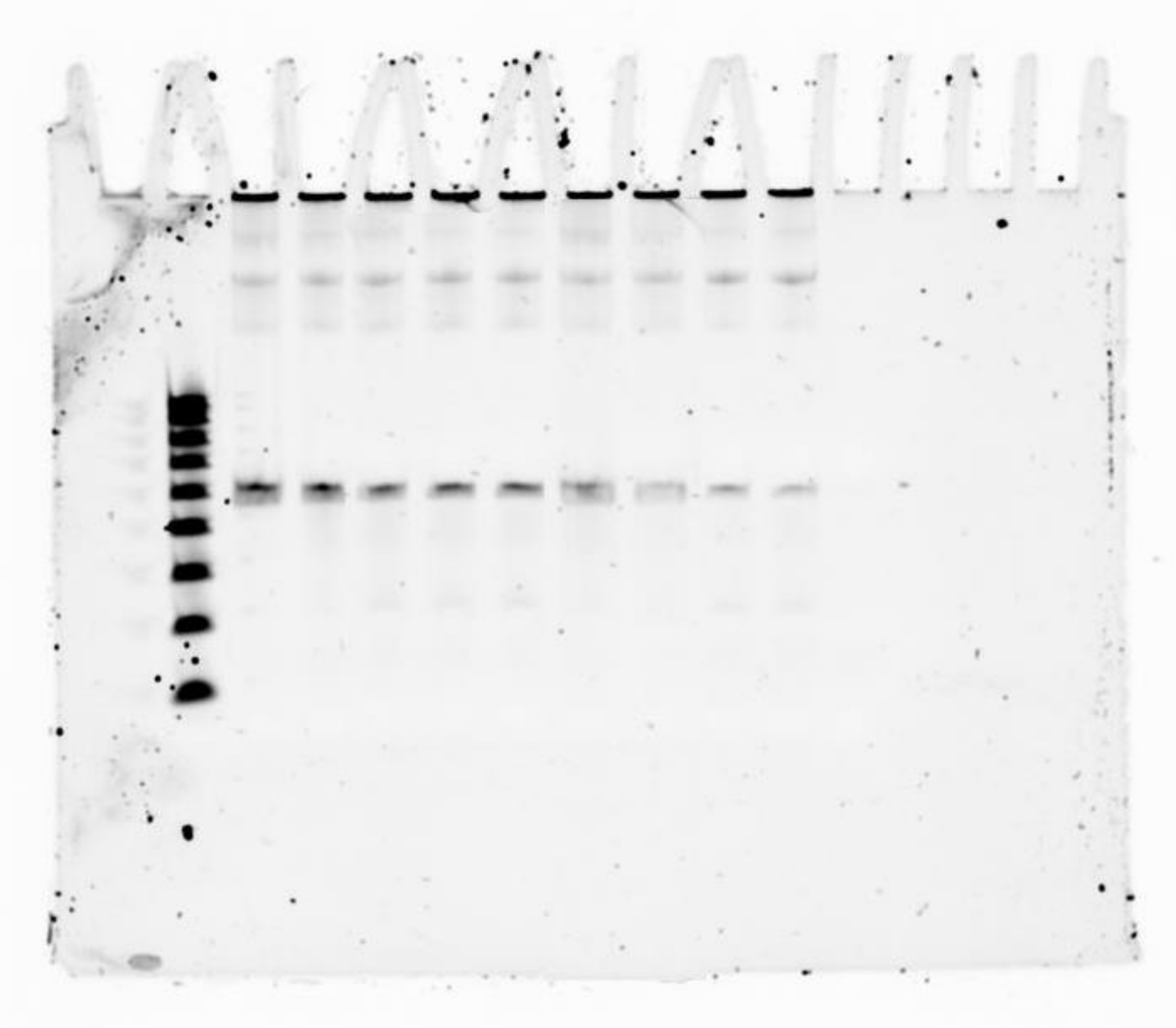

Figure 2B Annotated image of RC-ErbB2-idT

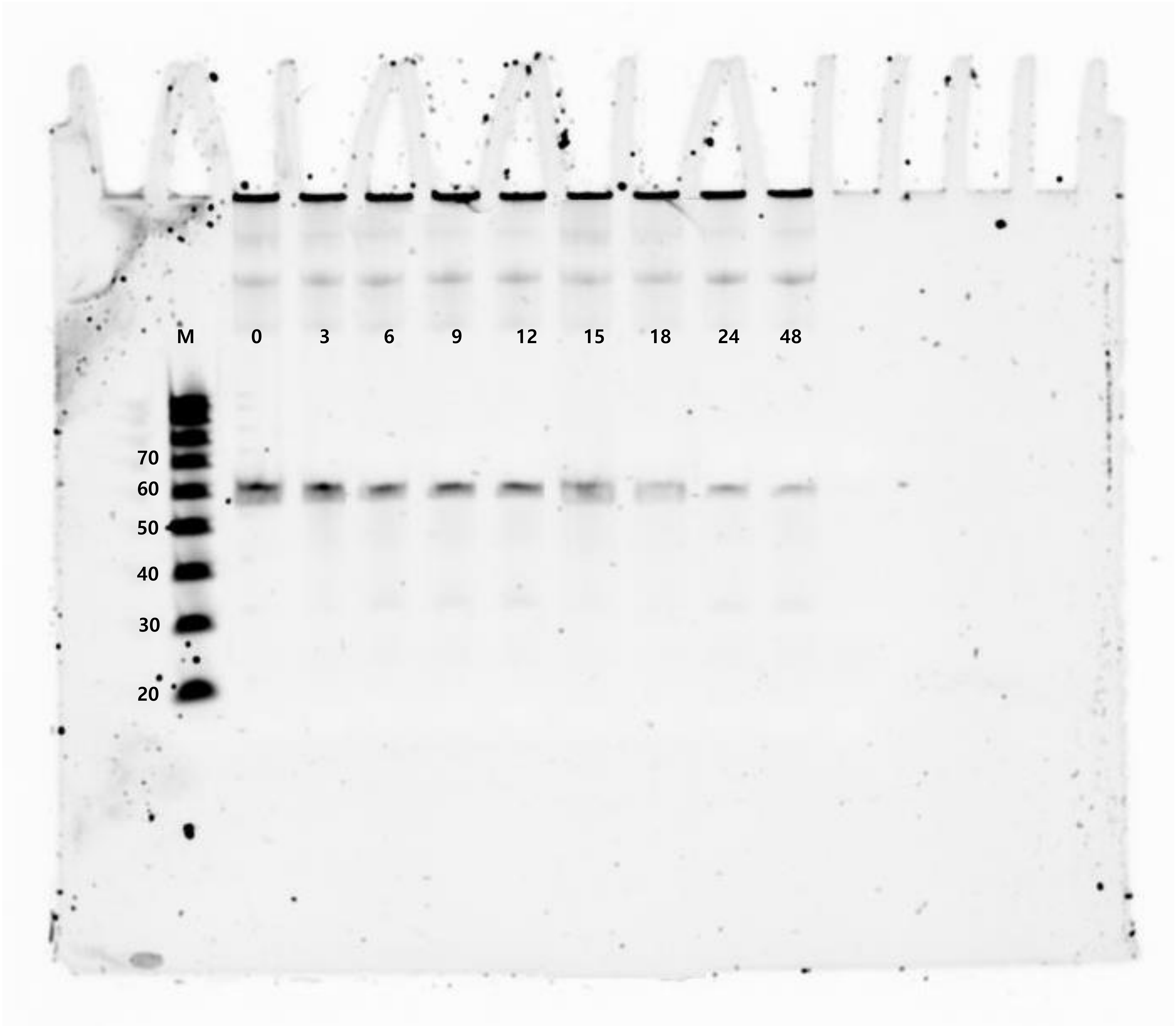

Figure 2B Original image of hyErbB2-idT

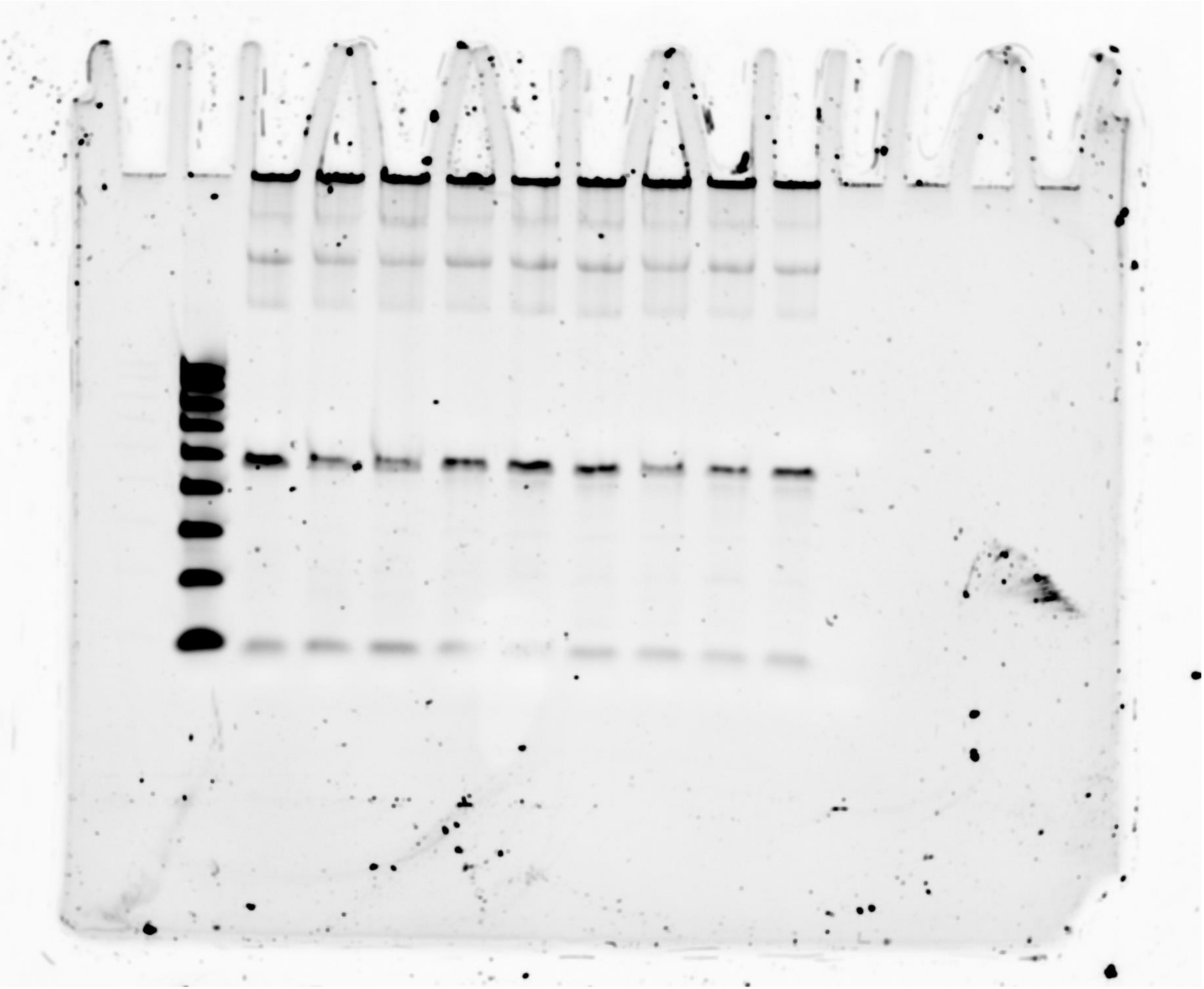

Figure 2B Annotated image of hyErbB2-idT

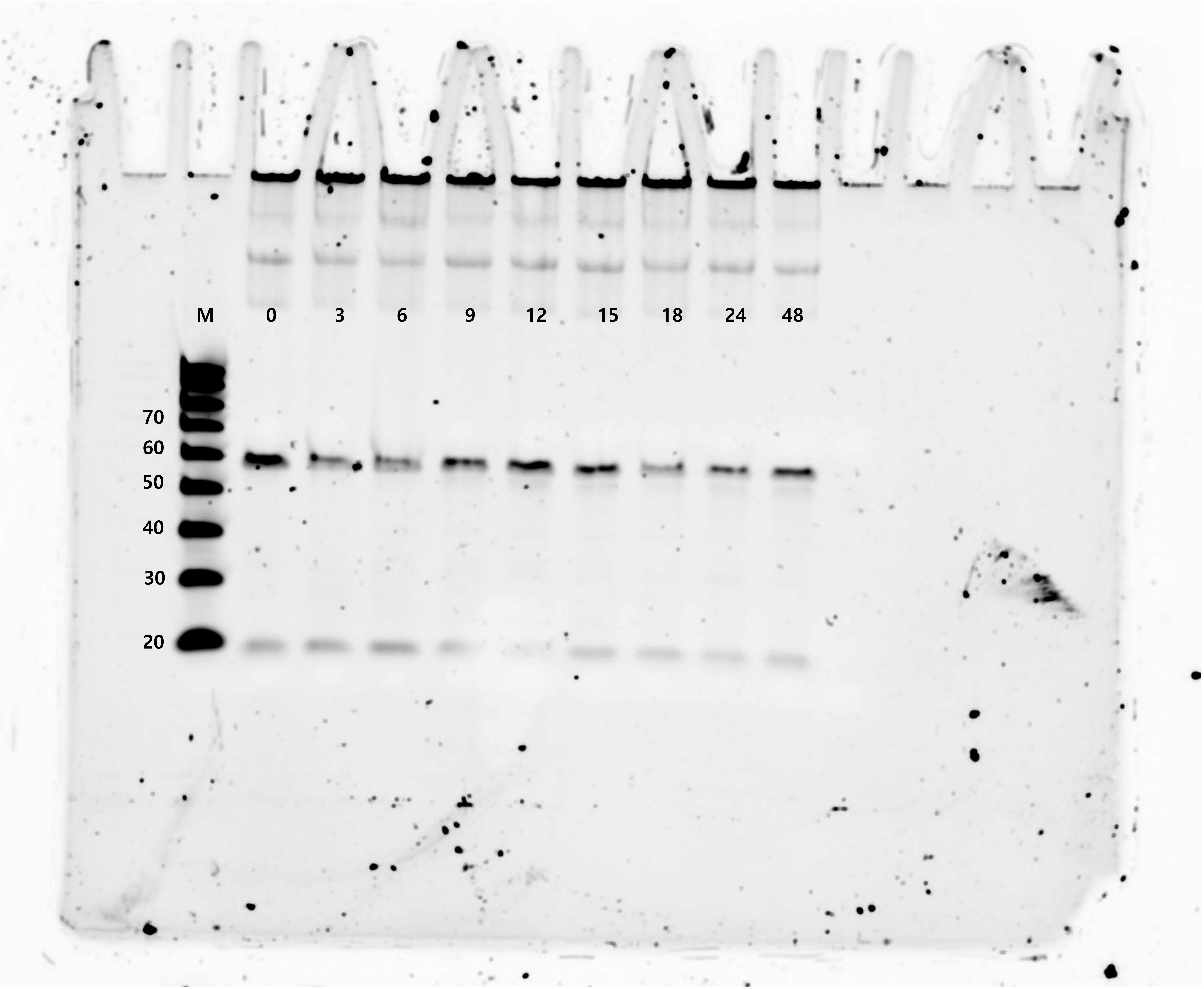

Supplement: S1 Raw images — (PDF) [file pone.0291624.s001.pdf]
