# Supplementary material for: Enhancement of in vivo targeting properties of ErbB2 aptamer by chemical modification
Source: PLoS One. 2023 Sep 20;18(9):e0291624. doi: 10.1371/journal.pone.0291624 (PMC10511116; doi:10.1371/journal.pone.0291624)
Supplement: Attachment — Submitted filename: Response to reviewers.docx [file pone.0291624.s002.docx]

**Manuscript PONE-D-23-11010**

**Response to Reviewers**

Dear Editor,

Thank you for giving us the opportunity to submit a revised draft of the manuscript “Enhancement of in vivo targeting properties of an ErbB2-specific aptamer via chemical modification.” for publication in the PLOS ONE. We appreciate the time and effort that you and the reviewers dedicated to providing feedback on our manuscript and are grateful for the insightful comments on and valuable improvements to our paper. We have incorporated most of the suggestions made by the reviewers. Those changes are highlighted within the manuscript. Please see below, in blue, for a point-by-point response to the reviewers’ comments and concerns.

**Reviewers' Comments to the Authors:**

Reviewer #1

The manuscript is evaluating the biological characteristics of the 3’-idT-modified ErbB2 aptamer (ErbB2-idT) compared with the unmodified aptamer via PET imaging using radioisotope F-18 labeling. The authors aim to improve stability and circulation time in blood for the aptamer by adding 3’idT modification. By preparing selective ErbB2 aptamer with a sequence (RC) having the 3’idT modification and a complementary sequence (cODN) with a radioisotope 18-F, the hybridized aptamer (18F-hyErbB2-idT) is used to study the ex vivo biodistribution and in vivo PET imaging in mice with ErbB2-expressing breast cancer (KPL4) xenografts. The 3’end modification alone did not completely prevent nuclease degradation but the idT-modified ErbB2 aptamer showed higher tumor uptake and longer blood circulation than unmodified aptamer in KPL4 xenografts which is of high relevance for development of future therapeutics. Moreover, the use of nuclear imaging to study in vivo biodistribution of modified nucleic acids seems to be a useful way of evaluating new modifications in future therapeutics. On this basis, I recommend the publication of this manuscript in PLOS ONE. Some minor points:

Author response: First, we would like to thank the reviewer for the detailed review of our manuscript. Most of the comments were accepted and the manuscript was revised according to the reviewers’ advices.

• p.10, line 19-20 (Introduction): State the biological function and relevance of ErbB2 in the introduction like in the first line in Results (“…erb-b2 receptor tyrosine kinase 2 (ErbB2, also known as HER2)…”).

Author response: We thank the reviewer for the suggestion. In accordance with the reviewers’ comments, we have added new paragraph in the revised manuscript: (As following)

“Erb-b2 receptor tyrosine kinase 2 (ErbB2, also known as HER2) is a member of the epidermal growth factor receptor family and plays an important role in regulating cell proliferation, survival, and differentiation [29]. ErbB2 is an attractive target for cancer diagnostics and therapy due to the overexpression in breast, gastric, lung, colon, ovarian, bladder, and gastroesophageal cancers [30].”

• p.16, line13 (Results): Write the full name of SELEX before abbreviation when used first time.

Author response: We thank the reviewer for the suggestion. In accordance with the reviewers’ comments, we have added the full name of SELEX in the revised manuscript.

• Figures are in general in low resolution. Figure 3A is difficult to read and see.

Author response: We thank the reviewer for the suggestion. In accordance with the reviewers’ comments, we have replaced low resolution Figures with a high-resolution Figures in the revised manuscript.

• p.29, Figure 1: Add a caption with 18F and Cy5 on picture.

Author response: Thanks for the comment. As per your suggestion, we have added figure caption for ^18^F and Cy5 in Figure 1.

• p.30, Figure 2B: Indicate the size of each M band on figure or clarify in figure text.

Author response: Thanks for this precious advice. As per your suggestion, we have mentioned the size of each M band on the Figure 2B.

• p.21, line 5 + Figure 5: “Fig 5. … The white arrows indicate location of the tumor." There are no white arrows on Figure 5. Please add this to the figure.

Author response: We apologize for these mistakes in our manuscript. As per your suggestion, we have added the white arrows on Figure 5.

Reviewer #2

The manuscript entitled "Enhancement of in vivo targeting properties of an ErbB2-specific aptamer via chemical modification" by Won Jun Kang is dedicated to the chemical stabilization of the aptamer. Author used inverted dT at 3'-end - the modification known since late 80s. Some stabilization was observed both in vitro and in vivo, however the lack of a control does not provide a chance to evaluate the real input of 3'-idT. Double stranded hyErbB2 w/o idT should be studied in parallel to clarify the input of the additional duplex in stabilization. Statistical analysis with p-values of fig 4 will provide the answer if idT modification lead to any statistically significant improvements. Now this point is not obvious. Also description of fig. 5 "The white arrows indicate the location of the tumor" should be removed or arrows should be added. The manuscript cannot be accepted in the current form and should be resubmitted after major revision.

Author response: We would like to thank the reviewer for this valuable review of our manuscript. Most of the comments were accepted and the manuscript was revised according to the reviewers’ advices.

Since hyErbB2 was paired with 15 bases, it did not significantly affect the characteristics of ErbB2 aptamer such as Kd value or specificity to target protein. In particular, the *Ex vivo* biodistribution study revealed that the metabolic patterns were different between ^18^F-hyErbB2

aptamer (hyErbB2 w/o idT) and ^18^F-hyErbB2-idT. Different metabolic patterns mean different stability because hyErbB2 w/o idT aptamer can be easily broken by nuclease. When looking at the biodistribution data 30 minutes after administration of hyErbB2 aptamer, the ^18^F-hyErbB2 aptamer (hyErbB2 w/o idT use as control) was discharged in a much faster than the ^18^F-hyErbB2-idT. At 1 h after injection, the blood level of ^18^F-hyErbB2-idT aptamer was 1.06 ± 0.15% ID/g and 0.85 ± 0.11 %ID/g for ^18^F-hyErbB2 aptamer, indicating that ^18^F-hyErbB2 aptamer can be more rapidly eliminated from the blood than ^18^F-hyErbB2-idT aptamer. In this study, it is believed that the biodistribution study sufficiently showed the difference in stability before and after attachment of 3'-idT.

In accordance with the reviewers’ comments, we have added p-values across the groups in Figure 4. Thanks for this precious advice. We apologize for the mistake in Figure 5. Based on your comment, we have added the white arrows on Figure 5 in the revised manuscript. Thank you for your opinion.
