# Supplementary material for: Enhancement of in vivo targeting properties of ErbB2 aptamer by chemical modification
Source: PLoS One. 2023 Sep 20;18(9):e0291624. doi: 10.1371/journal.pone.0291624 (PMC10511116; doi:10.1371/journal.pone.0291624)
Supplement: Attachment — Submitted filename: Rebuttal letter_Response to Reviewers_2_Minor reveision.docx [file pone.0291624.s003.docx]

August 1, 2023

Kira Astakhova

Academic Editor

PLOS ONE

Dear Editor,

Thank you for giving us the opportunity to submit a revised draft of the manuscript “Enhancement of in vivo targeting properties of an ErbB2-specific aptamer via chemical modification.” for publication in the PLOS ONE. We appreciate the time and effort that you and the reviewers dedicated to providing feedback on our manuscript and are grateful for the insightful comments on and valuable improvements to our paper. We have incorporated most of the suggestions made by the reviewers. Those changes are highlighted within the manuscript. Please see below, in blue, for a point-by-point response to the reviewers’ comments and concerns.

Sincerely yours,

Won Jun Kang, M.D., Ph.D.

Department of Nuclear Medicine

Severance Hospital

Yonsei University College of Medicine

50-1 Yonsei-ro, Seodaemun-gu

Seoul 03722, Republic of Korea

**Reviewers' Comments to the Authors:**

Reviewer #2: Thanks a lot for multiple changes that you performed - the quality of the manuscript is increased.

Author response: We would like to thank the reviewer for the detailed review of our manuscript.

However addition of the duplex part contributes even more than 3'-modification.

Author response: We agree to this comment. As the reviewer said, we also think the hybridization with complementary oligonucleotide affected stability. However, our data have shown that the attachment of idT at the 3’-end of the hybridized aptamer leads to the increased *in vivo* stability compared with unmodified hybridized aptamer.

In my opinion, 1.06 ± 0.15% ID/g vs 0.85 ± 0.11 %ID/g is a statistically significant difference, but regarding applications - this result does not generate value.

Author response: We thank the reviewer for the suggestion. The radioactivity of ^18^F-hyErbB2-idT aptamer (1.06 ± 0.15% ID/g ) in the blood at 1 h post-injection was significantly higher (P < 0.05) than that of ^18^F-hyErbB2 aptamer (0.85 ± 0.11 %ID/g). We think this data meaning that 3’-idT modified aptamer exhibits more longer blood circulation time compared to the unmodified aptamer.

The enrichment in the tumor differs for in vivo and ex vivo data (30 min and 1h, Fig. 4) - please address this point in the discussion. Another issue - data for the scrambled control is absent in Fig. 4A.

Author response: Thanks for your suggestions. We have already mentioned in the Results section that there is a difference in tumor uptake by time: (As following)

“At 30 min after injection, the tumor uptake of ^18^F-hyErbB2 aptamer (2.04 ± 0.69 %ID/g) was higher than that of ^18^F-hyErbB2-idT aptamer (1.57 ± 0.20 %ID/g). However, the radioactivity of ^18^F-hyErbB2-idT aptamer (1.36 ± 0.17 %ID/g) in the KPL4 tumors was significantly higher (*P* < 0.05) than that of ^18^F-hyErbB2 aptamer (0.98 ± 0.19 %ID/g) at 1 h post-injection, suggesting that extended blood circulation of ^18^F-hyErbB2-idT aptamer led to enhanced tumor accumulation.”

Thanks for the comment.
