# Supplementary material for: Enhancement of in vivo targeting properties of ErbB2 aptamer by chemical modification
Source: PLoS One. 2023 Sep 20;18(9):e0291624. doi: 10.1371/journal.pone.0291624 (PMC10511116; doi:10.1371/journal.pone.0291624)
Supplement: Attachment — Submitted filename: Rebuttal letter_Response to Reviewers_2_Minor reveision_2.docx [file pone.0291624.s004.docx]

August 25, 2023

Kira Astakhova

Academic Editor

PLOS ONE

Dear Editor,

Thank you for giving us the opportunity to submit a revised draft of the manuscript “Enhancement of in vivo targeting properties of an ErbB2-specific aptamer via chemical modification.” for publication in the PLOS ONE. We appreciate the time and effort that you and the reviewers dedicated to providing feedback on our manuscript and are grateful for the insightful comments on and valuable improvements to our paper. We have incorporated most of the suggestions made by the reviewers. Those changes are highlighted within the manuscript. Please see below, in blue, for a point-by-point response to the reviewers’ comments and concerns.

Sincerely yours,

Won Jun Kang, M.D., Ph.D.

Department of Nuclear Medicine

Severance Hospital

Yonsei University College of Medicine

50-1 Yonsei-ro, Seodaemun-gu

Seoul 03722, Republic of Korea

**Reviewers' Comments to the Authors:**

Reviewer #2: Thanks a lot for multiple changes that you performed - the quality of the manuscript is increased.

Author response: We would like to thank the reviewer for the detailed review of our manuscript.

However addition of the duplex part contributes even more than 3'-modification.

Author response: We agree to this comment. As the reviewer said, we also think the hybridization with complementary oligonucleotide affected stability. However, our data have shown that the attachment of idT at the 3’-end of the hybridized aptamer leads to the increased *in vivo* stability compared with unmodified hybridized aptamer.

In my opinion, 1.06 ± 0.15% ID/g vs 0.85 ± 0.11 %ID/g is a statistically significant difference, but regarding applications - this result does not generate value.

Author response: We thank the reviewer for the suggestion. As the reviewer said, the values (1.06 ± 0.15% ID/g and 0.85 ± 0.11 %ID/g) are too small. However, it was not intended to compare the uptake values between ^18^F-hyErbB2-idTaptamer and ^18^F-hyErbB2aptamer in the blood after 1 hour of administration. Our data showed that after 30 min of intravenous injection, the blood radioactivity of ^18^F-hyErbB2-idT aptamer and ^18^F-hyErbB2 aptamer was 1.69 ± 0.10 %ID/g and 2.21 ± 0.19 %ID/g, respectively. At 1 h after injection, the blood level of ^18^F-hyErbB2-idT aptamer was 1.06 ± 0.15% ID/g and that of ^18^F-hyErbB2 aptamer was 0.85 ± 0.11 %ID/g. After 1 hour injection, the radioactivity of ^18^F-hyErbB2-idT aptamer measured in the blood was reduced by 30% compared to that measured at 30 minutes, but for ^18^F-hyErbB2 aptamer, it was reduced by 61.5%. So, we have revised the Discussion section as follows: (As following)

“However, the radioactivity of ^18^F-hyErbB2-idT aptamer in the blood decreased by 37% from 30 min to 1 h post-injection, while ^18^F-hyErbB2 aptamer decreased by 61.5%. These results suggest that 3’-idT modified aptamer exhibits more longer blood circulation time compared to the unmodified aptamer.”

Thanks for the detailed comment.

Another issue - data for the scrambled control is absent in Fig. 4A.

Author response: Yes, there is no scrambled control data in Fig. 4A. The reason why we didn't do the biodistribution study of ^18^F-hyScrErbB2 aptamer at 30 min post-injection is because of the perfusion effect or non-specific binding at 30 minutes. However, after an hour, most of the aptamers were removed after metabolism and only targeted aptamers were left on the tumor, so we could verify whether the ^18^F-hyScrErbB2 aptamer really played a role as control, so we performed the biodistribution only after an hour using ^18^F-hyScrErbB2 aptamer.
